# Cryoanalgesia as the Essential Element of Enhanced Recovery after Surgery (ERAS) in Children Undergoing Thoracic Surgery—Scoping Review

**DOI:** 10.3390/jpm14040411

**Published:** 2024-04-12

**Authors:** Sławomir Zacha, Jowita Biernawska

**Affiliations:** 1 Department of Pediatric Orthopedics and Oncology of Musculoskeletal System, Pomeranian Medical University in Szczecin, 70-252 Szczecin, Poland; 2 Department of Anesthesiology and Intensive Therapy, Pomeranian Medical University in Szczecin, 70-252 Szczecin, Poland; jowita.biernawska@pum.edu.pl

**Keywords:** cryoanalgesia, perioperative care, pain management, enhanced recovery after surgery, ERAS

## Abstract

This article aims to present cryoanalgesia as an inventive strategy for pain alleviation among pediatric patients. It underlines the tremendous need to align pain management with the principles of the enhanced recovery after surgery (ERAS) approach. The aim of the study was to review the patient outcomes of nerve cryoanalgesia during surgery reported with regard to ERAS in the literature. The literature search was performed using PubMed and Embase to identify articles on the use of cryoanalgesia in children. It excluded editorials, reviews, meta-analyses, and non-English articles. The analysis focused on the study methods, data analysis, patient selection, and patient follow-up. This review includes a total of 25 articles. Three of the articles report the results of cryoanalgesia implemented in ERAS protocol in children. The research outcome indicates shortened hospital stay, potential reduction in opioid dosage, and significant progress in physical rehabilitation. This paper also describes the first intraoperative utilization of intercostal nerve cryoanalgesia during the Nuss procedure in Poland, highlighting its effectiveness in pain management. Adding the cryoanalgesia procedure to multimodal analgesia protocol may facilitate the implementation of the ERAS protocol in pediatric patients.

## 1. Introduction

Any surgical intervention generates a stress response within the body in various aspects. 

The physiological changes associated with ageing are responsible for a patient’s increased risk of complications after surgery [1,2]. Comorbidities (heart and vascular diseases, impaired kidney function, obesity, or diabetes), poor nutritional status, and addictions are typical risk factors for adult population. Additionally, decreased reserve, impaired functional status, and low physical activity may be associated with higher complication rates. Preoperative anxiety, fasting, emotional distress, tissue damage, hypothermia, hypoxia, pain, hemorrhage, and fluid shifts are major stressors on organ systems. These aspects, due to homeostasis disturbances, lead to the “stress response” to the surgical procedure. The “stress response” is caused by metabolic and hormonal change, which results in inadequate immunological and endocrine activity. Stress-related activation of hypothalamic–pituitary–adrenal axis and an elevation of cortisol, catecholamines, and glucagon induce the predominance of a pro-inflammatory status. These mediators modulate the homeostatic state, which may lead to a generalized response: surgical stress-induced organ injury. Evidence suggests that this phenomenon, if left untreated, may lead to increased morbidity and mortality. It may explain the importance of optimizing perioperative care management to modify the physiological stress response and expedite the recovery process. 

The implementation of “fast-track” or “enhanced recovery pathway” integrated procedures into one evidence-based perioperative care protocol created the enhanced recovery after surgery (ERAS) approach. Integrated ERAS protocol has shown that hospital length of stay and complications can be reduced for many surgical procedures: bariatric, breast, cardiac, colorectal, gastrointestinal, gynecology, head and neck, liver, lumbar spinal fusion, neonatal, obstetrics, orthopedic, thoracic, urology, and vascular surgeries. The guidelines for various surgical procedures differ only slightly. Key points include pre-, intra-, and postoperative interdisciplinary activities.

Implementing an ERAS program requires a multidisciplinary approach. The role of each member of the team in implementing ERAS protocol is described widely in the literature. The roles of anesthesia techniques, surgical procedures, perioperative care, and post-surgery rehabilitation in reducing complications and shortening hospitalization time are crucial. Several ERAS interventions supervised by an anesthesiologist may reduce surgical stress. Preoperative education may mitigate mental stress and reduce the demand for premedication. Preoperative carbohydrate loading, no prolonged preoperative fasting, and early postoperative feeding modulate and mitigate the metabolic response (e.g., insulin resistance). The maintenance of fluid and electrolyte balance, prevention of hypothermia, and prevention of nausea and vomiting may improve tissue perfusion and prevent the consequences of hypotension. Adequate short-acting anesthesia and well-controlled analgesia protect patients from pain-inducted consequences: mental and physical stress response and delayed mobilization and rehabilitation. The use of multimodal analgesia, including regional techniques, is well proven and recommended by guidelines both in adult and in pediatric populations. 

The adequate use of antibiotic prophylaxis and thromboprophylaxis protects patients from wound complications. Surgical activities for reduced complications, namely minimally invasive surgical techniques and the early removal of the catheter and drains, are well documented in the literature. Depending on the perioperative care, the patient’s recovery time in the hospital may unnecessarily increase. The avoidance of complications is the most important factor for shortening hospitalization time. By optimizing perioperative care management, the patient’s physiological response to stress induced by surgery can be modified and, therefore, the recovery process shortened. Integrated efforts in all aspects, e.g., anesthesia techniques, surgery, care, and post-surgery rehabilitation, can effectively reduce the risk of unnecessary complications. This directly translates into both hospitalization time and overall treatment costs. The concept of enhanced recovery after surgery (ERAS) protocol aims to optimize the patient’s condition by shortening the overall recovery period (http://www.erassociety.org, accessed on 1 March 2024) [1]. 

The application of ERAS protocol has well-documented results in surgery, mainly in the adult population [3,4,5,6]. However, the research outcome within the pediatric group is limited. Therefore, there is a need for further exploration of the efficacy and feasibility of ERAS protocol implementation in pediatric surgical settings. The effective management of acute postoperative pain is crucial for the extensive implementation of the ERAS protocol. Successful pain control can improve a patient’s comfort, enhance rehabilitation effectiveness, reduce complication risks, and shorten hospitalization time, ultimately decreasing the total cost of treatment.

Thoracic surgery commonly induces severe and persistent pain that can last for several weeks following the procedure. Clinical studies have confirmed that patients who endure unacceptable levels of acute postoperative pain, along with experiencing significant complications due to pain, are more likely to report ongoing discomfort at both the 30-day and 1-year marks, attributed to a dysregulated inflammatory response [7,8]. The risk of this pain becoming chronic exceeds thirty percent [9]. Currently, the recommended approach for managing acute pain and preventing its transition to chronicity involves multimodal analgesia. This strategy entails using various analgesics with differing mechanisms of action and employing diverse administration techniques [10]. Given the intricate innervation of the chest wall, a consensus on a single effective regional pain relief method remains elusive. However, commonly favored techniques for regional analgesia include thoracic epidural anesthesia, paravertebral block, intrapleural block, fascial block of the dorsal extensor muscle compartment (known as erector spinae plane or ESP block), or multilevel intercostal nerve blocks. The implementation of regional analgesia is crucial due to its associated benefits, such as reduced pain scores, decreased reliance on opioids, faster patient mobilization and independence, improved respiratory function and sleep quality, as well as lower morbidity and mortality rates. Practitioners may choose between a one-time administration or continuous anesthesia via catheter placement. Because of the lack of consensus on optimal pain management strategies, cryoanalgesia is important and has a potential role in this context.

Following the principles of multimodal analgesia, the diagnostic and treatment plan for pain should be individually adjusted according to the patient’s age and capabilities. Because of the way different drugs work, various drugs should be utilized and appropriate administration techniques applied. For certain procedures, pharmacotherapy itself may be insufficient. Therefore, there is a need to seek methods to improve the patient’s comfort and expedite their return to full mobility. The application of cryoanalgesia to peripheral nerves during the perioperative period is one of such non-pharmacological methods that leads to a temporary blockage of pain conduction. It improves the quality of recovery and hence fulfills the ERAS protocol requirements. 

The goal of this study was to determine whether incorporating cryoanalgesia to the multimodal analgesia during the peri-operative period in children may be added to ERAS protocol.

## 2. Materials and Methods

The methodology of this study follows the rules of “scoping review” methods by Arksey and O’Malley that include the following [11]:

1. Specifying the research question: Can cryoanalgesia combined with multimodal analgesia during the peri-operative period in children improve the effectiveness of ERAS protocol?

Regarding numerous scientific reports on the effective use of cryoanalgesia in adults, the authors aimed to analyze the current body of knowledge in this matter among pediatric patients. The assumptions of multimodal cryoanalgesia include the possibility of multidirectional slowdown of nociception by incorporating various techniques and synergistic pharmacotherapy. Therefore, the possibility of applying cryoanalgesia for peripheral nerves in order to induce temporary pain blockage fits the principle. ERAS protocol is used for optimization of the recovery process after the surgery. Effective pain control allows efficient rehabilitation after surgery, improves the patient’s comfort, prevents any potential complications, and shortens hospital stay.

2. Identifying relevant literature.

Both authors analyzed the data in PubMed and Embase databases with the use of keywords such as “cryoanalgesia”, “enhanced recovery after surgery, ERAS”, and “children, adolescent, pediatrics”. A manual search was then performed on the references in relevant publications focused on the use of cryoanalgesia in children during the peri-operative period regarding ERAS protocol.

3. Selection of studies.

**Figure 1:**
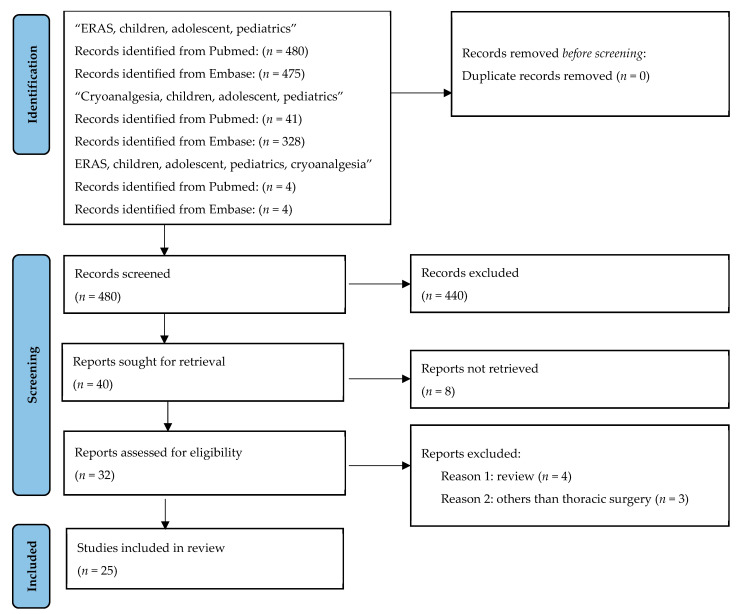
PRISMA chart.

Both authors participated in the selection of studies. We applied inclusion criteria: The search focused on English publications, regardless of the publication date. The details are described below in Figure 1.

4. Data evaluation.

Each study followed the performance evaluation scheme in terms of group size, study type (prospective or retrospective), randomization, surgery length, hospital stay length, postoperative pain intensity, amount of opioids used during the postoperative period, early and long-term observation time, and reported complications.

5. Summarizing, synthesizing, and describing the results.

The study presents the case history, physical methods, and histological results in order to introduce the reader to the topic and clinical use. 

## 3. Results

ERAS protocol application in children is well described in 480 articles in PubMed and 475 articles in Embase, mainly regarding scoliosis correction, abdominal procedures, and oncological surgeries [12,13,14,15,16]. ERAS protocol in the field of pediatric thoracic surgeries when cryoanalgesia was used is described in four articles only (one review and three original) [17,18,19,20]. 

From a total of 41 articles from PubMed and 328 articles from Embase that described the use of cryoanalgesia in the pediatric population, 25 articles were included in this review. The details are depicted in Figure 1—PRISMA chart. All of the articles describe the utilization of intercostal nerves before or during thoracic surgery in children. 

Three articles reported the results of cryoanalgesia implemented in ERAS protocol. Two of them originated from Poland (Department of Pediatric Orthopedics and Oncology of Musculoskeletal System, Pomeranian Medical University, Szczecin). Both of these described how to adapt the ERAS protocol for adults to the specifics of pediatric orthopedic conditions and implement cryoanalgesia for pain control [17,18]. DiFiore et al. described next-day discharge after the Nuss procedure using intercostal nerve cryoanalgesia and peri-operative ERAS pain protocol [19]. 

One may ask why cryoanalgesia should be added to multimodal analgesia in children. Pharmacotherapy is insufficient in many cases. Adjunctive methods are needed to improve patient comfort, safety, and recovery, according to the idea of ERAS [21,22,23,24].

Why should we use cryoanalgesia?

### 3.1. Foundations of Cryoanalgesia: Physical and Structural Aspects

The application of low temperatures in medicine has been employed since the time of Hippocrates [25]. In the 1970s, probes utilizing the Joule–Thomson phenomenon were constructed. There are two concentrically arranged channels of different diameters within these probes. As gas flows through a porous partition from an area of smaller diameter (higher pressure) to an area of larger diameter, rapid gas expansion (pressure drop) occurs. This results in a significant temperature decrease at the tip of the probe to approximately −80 degrees Celsius. Currently, nitrous oxide or carbon dioxide is commonly used [26,27].

After the application of low temperature, osmotic shifts of water and the formation of intracellular and extracellular ice crystals are observed. Depending on the freezing time and tissue hydration, the area subjected to negative temperature change varies. As a result, reversible axonal damage occurs. Histological studies have shown that, according to Seddon, cryoanalgesia causes axonotmesis damage (Grade II, according to Sunderland). This is a temporary damage to the axons while maintaining integrity within the perineurium, epineurium, and endoneurium. Unaffected blood vessels and the myelin sheath along with the nerve environment cytokines and growth factors determine nerve regeneration along the Keya–Retzius sheath. Transient damage to nerve fibers temporarily blocks conduction and leads to loss of function: pain, cold, touch, movement, or autonomic activity. After structural regeneration of the axons, physiological functions are restored within a few to several weeks. The local autoimmune effect of cryoanalgesia eliminates pathological receptor sites, minimizing the risk of pain conversion to a neuropathic form and the formation of neuromas [28,29].

### 3.2. Initiation and Duration of Cryoanalgesia

The clinical analgesic effect following cryoanalgesia application varies over time. The effect of blocking impulse conduction in nerve fibers can be compared to that of a locally anesthetic drug intake. However, the prolonged effect is due to the formation of micro-crystals, leading to swelling and a subsequent reversible loss of function. The key advantage of freezing over other non-pharmacological pain relief methods is its extended effectiveness, lasting two months at minimum, and the gradual return of sensation [26,27,28,29].

### 3.3. Duration of Cryoanalgesia Application

The studies show that a standard duration of application during intercostal nerve freezing was 2 min. However, Zeineddin et al., in their study evaluating the benefits of using a shorter application time, demonstrated that applying low temperature to the intercostal nerve for even one minute can already achieve the previously described benefits [30].

### 3.4. Clinical Application

The use of cryotherapy in the treatment of acute and chronic pain is prevalent, particularly among adult patients in various medical domains. Cryotherapy has proven effective in pain treatment of rheumatic etiology, metabolic origins, and in dermatology, gynecology, ophthalmology, otolaryngology, and cardiology. It is successfully employed in trauma surgery, orthopedics, neurology, and oncology [31,32]. Cryotherapy procedures are performed under the guidance of real-time ultrasound or fluoroscopy [33,34,35,36]. Since any surgery performed within the chest area generates intense discomfort, cryoanalgesia of intercostal nerves in thoracic surgery is exceptionally relevant and thus well documented in the adult population. Sepsas et al. conducted a randomized study to compare the effects of cryoanalgesia combined with intravenous morphine in patients undergoing thoracotomy. They assessed the intensity and the incidence of post-thoracotomy pain, numbness, epigastric distension, back pain, the analgesic requirements. They concluded that cryoanalgesia may be considered a simple, safe, inexpensive, long-term method of post-thoracotomy pain relief [37]. Park et al. supported these results [38]. 

For this reason, the use of cryoanalgesia for thoracic procedures in children is promising. Lai et al. reviewed the pediatric results of studies describing the Nuss procedure or minimally invasive repair of pectus excavatum. The authors concluded that intercostal nerve cryoanalgesia effectively controls pain, decreasing postoperative opioid use and hospital length of stay with few short-term complications [20]. However, the literature on the use of cryoanalgesia in the pediatric population is limited to thoracic surgical procedures only [39,40,41,42,43]. Its primary indication is for the treatment of acute pain and the prevention of chronic pain following corrective surgeries for anterior chest wall deformities. 

Contraindications to the procedure include acute systemic infections and clinically significant coagulopathies.

### 3.5. Application of Cryoanalgesia in Children

The gold standard for the surgical correction of funnel chest deformities is the minimally invasive Nuss technique using thoracoscopy. An alternative is the Ravitch method performed using an open approach. Chest surgeries cause significant pain due to the complex innervation of the chest wall. Whereas there is no consensus on a single effective method of pain management, it is clear that in case of intravenous multimodal analgesia with regional anesthesia techniques applied, postoperative pain control is insufficient [33,34,35,36,37,38,39,40,41,42,43,44].

The first reports on the use of cryoanalgesia during chest wall deformity correction surgeries date back to 2016. Kim et al. and Keller et al. described lasting relief in pain control after a single application of cryoanalgesia during surgery, providing more effective analgesia to the anterior chest as compared to the use of extrapleural catheter anesthesia [45,46]. This allows for a reduction in total opioid dosage and hospitalization time. Graves et al. presented promising results from a randomized study comparing the effects of cryoanalgesia and extrapleural catheter anesthesia in pain management after the Nuss procedure [47]. Freezing nerves significantly shortened length of hospital stay and reduced postoperative opioid intake as compared to extrapleural anesthesia in the chest area. Despite a significant reduction in opioid consumption with cryoanalgesia, both methods provide equally effective pain control.

Dekonenko et al. compared pain treatment methods using extrapleural anesthesia, patient-controlled analgesia, and cryoanalgesia of intercostal nerves [48]. The results indicated benefits in terms of a faster initiation of oral analgesic use and the duration of their application. According to the authors, the use of cryoanalgesia significantly prolonged the operation time. Results from the national registry by Arshad et al. showed that cryoanalgesia was associated with a shorter hospital stay without an extension of operation time and anesthesia duration as compared to procedures involving extrapleural or epidural anesthesia prior to surgery [49]. Short-term complication rates were similar in both groups. Furthermore, the importance of one-lung ventilation with selective intubation for optimal visualization during cryoanalgesia of intercostal nerves was emphasized. Results from Pecoraro et al., Cadaval et al., Lai et al., Hegde et al., and Arshad et al. in a larger study group confirmed the benefits of cryoanalgesia [40,49,50,51,52]. Toselli et al. highlighted technical considerations for optimizing the “freezing” procedure [41]. Cryoanalgesia should be performed before lifting the sternum and implanting the brace, as this is advantageous for controlling postoperative pain caused by chest wall distortion and microfractures. Placing the cryoprobe in the middle of the intercostal space with a cephalad pressure and 1 cm medial to the anterior axillary line ensures optimal contact with the target nerve, minimizing the risk of lung damage. Talsma et al. described the presence of connecting branches between adjacent intercostal nerves [53]. Sectional studies confirmed anatomical diversity in the departure of the lateral connecting branch and its passage through intercostal muscles.

The beneficial effects of cryoanalgesia have also been confirmed in cases of chest deformities other than funnel chest. Pilkington et al.’s study compared cryoanalgesia of intercostal nerves with extrapleural anesthesia in the chest area after modified Ravitch procedures [54]. In the group of patients subjected to cryoanalgesia, hospitalization time was shortened by two days as compared to patients receiving extrapleural analgesia.

### 3.6. Intraoperative Intrathoracic and Preoperative Percutaneous Technique

The intraoperative procedure during thoracoscopy enables precise localization of the cryoprobe, and the patient undergoes general anesthesia only once. However, it should be noted that the final effect of cryoanalgesia will be achieved after several days, during which bridging treatment for acute postoperative pain should be applied. Performing the “freezing” procedure in advance (before surgery) using a percutaneous probe requires additional general anesthesia for the patient. Visualization of the probe’s position is indirect, using ultrasound imaging or nerve stimulation. Velayos et al. were the first to present results using percutaneous cryotherapy 48 h before the Nuss procedure [55]. The authors reported lower pain intensity, reduced opioid consumption, and shorter hospitalization time as compared to cryoanalgesia applied on the day of the surgery.

Until now, nerve-“freezing” procedures during surgery have been employed in the USA and several European countries [39,40,41,42,43]. In Poland, cryoanalgesia among children was performed for the first time during the Nuss procedure in May 2022 at the Department of Pediatric Orthopedics and Oncology of the Musculoskeletal System, Clinical University Hospital No. 1 of the Pomeranian Medical University, Szczecin. The Cryo-S Painless device from the Polish company Metrum Cryoflex (Warsaw, Poland) along with the A-30/300/PEA/R/RF cryogenic probe were used for cryoanalgesia during thoracoscopy. Until that time, cryoanalgesia had only been applied in Poland among adults. Our research results confirm the previous observations [18]. In comparison to standard multimodal analgesia, intraoperative “freezing” allows for more effective relief of acute postoperative pain, reduction in opioid usage, more efficient rehabilitation at an early stage, and shortened hospitalization time. Therefore, it enables the achievement of goals for which the ERAS (enhanced recovery after surgery) protocol was created [17].

### 3.7. Study Endpoints for Cryoanalgesia

Cryoanalgesia is considered safer than chemical or high-temperature neurolysis. Only half of the articles describing cryoanalgesia reported the frequency and type of complications. No differences or significantly fewer complications were found in the cryoanalgesia group as compared to patients anesthetized conventionally [19,39,56,57,58,59,60,61,62,63,64,65,66,67,68,69,70]. The reduction in complication frequency mainly pertained to urinary retention, a common adverse effect of opioid use [69,70].

An important aspect of assessing postoperative pain is informing the patient about the need to distinguish increased tension in the chest due to sternum and rib elevation from actual postoperative pain. Studies have not yet evaluated the risk of chronic pain in the pediatric patient group subjected to cryoanalgesia.

Regarding complications arising from cryoanalgesia, the occurrence and duration of sensory disturbances such as hyperesthesia or hypoesthesia were assessed. Only 16% of studies analyzed the outcome of superficial sensory disturbance diagnostics after “freezing” [17,19,30,35,58,70]. A one-year follow-up research demonstrated complete sensory function restoration in 76–100% of patients. No pediatric patients experienced neuropathic pain within the twelve-month observation period [26,30]. None of the articles provided a detailed description of the adopted sensory disturbance diagnostic method. The Graves et al. research group’s observation is intriguing [26]: Researchers found that all ten patients had sensory disturbances 14 days after cryoanalgesia during the Nuss procedure. Simultaneously, 20% of patients without cryoanalgesia also exhibited sensory disturbances of the same intensity. The authors concluded that this complication may result from the surgical technique itself and not from the cryoanalgesia. Additionally, sensory function improved or normalized in all patients within a year after the procedure.

Currently, it is acknowledged that hyperesthesia or hypoesthesia of skin innervated by nerves subjected to cryoanalgesia occurs in less than 1% of cases [24]. In safety studies comparing the discussed procedure to other analgesic strategies in adults, there was no increased incidence of neuropathic pain. However, in the pediatric patient group, the frequency of neuropathic pain one year after the procedure was 0% [26,27].

In case of continuous analgesia through an extrapleural catheter, the intensity of acute postoperative pain is well controlled only during the action of local anesthetics. Therefore, after 72 h, following the end of regional analgesia, patients suddenly experience very strong pain, which typically requires prolonged opioid use. The application of cryoanalgesia allows for a reduction in acute postoperative pain intensity and significantly lower visual analog scale (VAS) scores [26,43,51,57,60,61,62,63,64]. Pain intensity reduction leads to less consumption of potent analgesics, including opioids. All studies comparing the effectiveness of cryoanalgesia to other pain management strategies confirmed its positive effect [19,24,30,46,51,59,60,61,62,63,64,65,66,67,68,69,70]. This enabled reduction in opioid dose and duration of its use during hospitalization. Considering the global opioid abuse trend, Zeineddin et al. demonstrated that by incorporating cryoanalgesia into surgery, postoperative opioid use during hospitalization can be reduced. The authors emphasized the impact of these aspects on the total cost of treatment. Study limitations include a retrospective nature, differences in analgesic protocols, and exclusion of patients with more than one plate implantation [30].

The analysis also considered the occurrence of unintended intraoperative hypothermia resulting from cryo application and an increased risk of postoperative wound infections. Bundrant et al. showed that undergoing cryoanalgesia did not show a connection with postoperative wound infections [36]. Only one study did not confirm a significant reduction in hospitalization time after cryoanalgesia as compared to extrapleural anesthesia in the chest area [57]. These outcomes may be due to the very small number of patients undergoing nerve-freezing analgesia (six individuals). All other studies assessing the length of hospitalization after cryoanalgesia compared to any other pain treatment method (regional anesthesia or continuous intravenous analgesia) confirmed its significant reduction. Some researchers showed that when regional anesthesia is added to a “freezing” procedure, pain control allows for hospital leave on the first day after surgery [19,30,34,48,50,58,59,60].

Keller et al. in 2016 and Sun et al. in 2021 observed an increased incidence of plate migration requiring reoperation in up to 12% of patients undergoing cryoanalgesia during surgery. According to the authors, this was due to the possibility of more intense rehabilitation and increased patient activity [46,68]. The largest study on patients operated using the Nuss technique did not confirm these observations and reported plate dislocation in less than 1% of patients [43,63,67].

### 3.8. Cryoanalgesia as the Essential Element of ERAS in Children

Zacha et al. evaluated the effectiveness of intercostal nerve cryoanalgesia in terms of pain management in relation to the routinely used multimodal analgesia in children during the Nuss procedure. Authors also described the impact of using the proprietary “BackOnFeet” application as a part of patients’ education of ERAS on the quality of life of patients after surgery. It was a prospective, single-center, non-randomized, before–after pilot study. The authors concluded that intraoperative intrathoracic cryoanalgesia of intercostal nerves is more effective in relation to the routinely used multimodal analgesia. The cryoanalgesia procedure allowed the shortening of the duration of opioid use and hospitalization time, improved quality of postoperative rehabilitation, and enabled ERAS protocol introduction. Moreover, the use of the proprietary “BackOnFeet” application had a positive effect on the quality of life of patients after surgery [18]. Cryoanalgesia performed during thoracic surgery optimized pain management and facilitated early rehabilitation and hospital discharge. These aims were achieved in the “before–after” observational study. Authors concluded that hospitalization time as a result of protocolized care and the use of intraoperative cryoanalgesia within the “after” group was reduced from six to three days [17]. 

DiFiore at al. conducted a prospective study that confirmed the essential hospitalization time reduction when cryoanalgesia was added to the ERAS–pain protocol. Authors concluded that INC combined with bupivacaine intercostal nerve blocks and a pre- and post-hospital analgesia protocol facilitated discharge one day after the Nuss procedure, achieved excellent pain control, and eliminated the need for intravenous opioids [19].

## 4. Discussion

The application of ERAS protocol within the pediatric group is limited. It is mainly focused on ERAS protocol application in scoliosis correction, abdominal procedures, and oncological surgeries [12,13,14,15,16]. The available literature does not describe wide ERAS protocol use in the field of pediatric thoracic surgeries. Implementation of ERAS adapted to local conditions was described in three articles only [17,18,19].

The cryoanalgesia of intercostal nerves, utilizing low temperatures in the treatment of postoperative pain among children, is gaining increasing popularity. It allows pain relief for several months, and with regular pain intensity assessments and multimodal analgesia, cryoanalgesia aligns with the ERAS protocol. Among children operated for various chest deformities, this procedure is most often performed intraoperatively during thoracoscopy and single-lung ventilation. Cryoanalgesia applied during the Nuss procedure is now recognized as a standard pain management strategy by the Chest Wall International Group (CWIG). 

In the current literature, most studies were performed retrospectively. However, all the studies conducted prospectively were possibly underpowered due to a small sample of patients. Among the prospective studies, cryoanalgesia was only compared to thoracic epidural catheter, opioids infusion, and intercostal nerve blocks. There has only been one randomized control trial comparing cryoanalgesia with any other analgesic strategy. For these reasons, this scoping review has some limitations. However, the main limitation of the present study is due to the small number of studies that describe the main topic. It is possible that some studies are not available for open access. The idea of ERAS emphasizes some strategies that enable shortening hospitalization time and lessening complications. The inclusion of cryoanalgesia into multimodal analgesia may thus facilitate perioperative pain management.

## 5. Conclusions

Cryoanalgesia is considered a valuable addition to pain management strategies. The current article underlines the need for ongoing research and a comprehensive, multidisciplinary approach to maximize its benefits. Adding the cryoanalgesia procedure to a multimodal analgesia protocol may facilitate the implementation of the ERAS protocol in pediatric patients. However, this topic is not sufficiently described in the literature. 

## Data Availability

The raw data are available upon request from the corresponding author.

## Abbreviations

ERAS	Enhanced recovery after surgery
VAS	Visual analog scale
CWIG	Chest Wall International Group
